# Assessing student paramedic visual and verbal checks for defibrillation safety—an observational study

**DOI:** 10.1186/s40064-015-1570-x

**Published:** 2015-12-14

**Authors:** Malcolm J. Boyle, Brett Williams, Linda Ross

**Affiliations:** Department of Community Emergency Health and Paramedic Practice, Monash University, Building H, McMahons Rd, Frankston, VIC 3199 Australia

**Keywords:** Heart arrest, Emergency medical technician, Defibrillation, Safety

## Abstract

One of the cornerstones in resuscitation training is defibrillation safety, inadvertent “shocking” of the patient when another person has contact with the patient may have a range of safety consequences. The objective of the study was to assess visual and verbal safety checks by paramedic students prior to defibrillation. This was a prospective observational mannequin study of defibrillation safety during a simulated cardiac arrest by paramedic students. The study was conducted in the lounge room of the Department of Community Emergency Health & Paramedic Practice simulation flat, a replica of a complete flat where prehospital simulations are conducted. Each student completed two 10-min cardiac arrest simulations with multiple defibrillation attempts. Each student and an independent Faculty member rated the simulation safety performance using a defibrillation safety self-assessment (DSSA) form. Twenty-four (20 %) students participated in the study with 14 (58 %) being female. For scenario one agreement between student and assessor proved significant for “scanning the incident scene” for all three defibrillation attempts, with agreement ranging from 29 % (p = 0.044) to 47 % (p = 0.007), and stating “stand clear” for defibrillation attempt one and three with the agreement ranging from 47 % (p = 0.007) to 100 % (p < 0.001). For scenario two agreement between student and assessor proved significant for “charging eye contact” for all three defibrillation attempts, with agreement ranging from 40 % (p = 0.043) to 53 % (p = 0.003), and “scanning the scene to ensure all persons are clear of the patient” before defibrillation attempt one and two with agreement ranging from 29 % (p = 0.044) to 46 % (p < 0.007). The results of this study suggest student perception of their performance and what they actually do is vastly different. Further studies using video recording glasses are required so students can gain an accurate and realistic sense of their defibrillation safety performance.

## Background

Defibrillation is an integral component of the “chain of survival” for a patient in cardiac arrest and is used by first responders through to various levels of healthcare professionals (Cummins et al. 1991; Nolan et al. 2010). One of the emphasises in resuscitation training is defibrillation safety; inadvertent “shocking” of the patient when another rescuer or bystander who has contact with the patient may have a range of potentially harmful consequences, the worse being sending the rescuer or bystander into cardiac arrest.

Resuscitation training involving defibrillation is focussed on safety for the rescuers, bystanders and the patient. During the analysis of the patient’s heart rhythm by the defibrillator and immediately prior to “shocking” the patient it is the responsibility of the lead rescuer to ensure no rescuers, bystanders or equipment is touching the patient (Kerber 2008). This scene safety checking is done both verbally and visually; it includes looking around the patient making sure no one is touching the patient and making visual contact with other rescuers to ensure they understand the commands. The verbal commands make sure that all rescuers and bystanders are aware of what is happening and other rescuers confirming they and the bystanders, are “clear” of the patient prior to the “analysis” of the heart rhythm and immediately prior to pressing the shock button.

There have been previously documented cases where a rescuer has received a “shock” during the resuscitation process (Gibbs et al. 1990). The majority of these incidents were due to a deviation in the process ensuring safety prior to “shocking” the patient. Therefore resuscitation training processes involving defibrillation safety should take great precedence. There is often disagreement between the training participant and facilitator in relation to various aspects of safety performance, unless the training session is recorded and reviewed.

We have not identified any previous studies that have assessed paramedic student visual and verbal safety during simulated resuscitation, especially when defibrillation is used. The objective of the study was to assess visual and verbal safety checks by paramedic students prior to defibrillation.

## Methods

### Design

This was a prospective observational mannequin study of defibrillation safety during a simulated cardiac arrest by paramedic students.

### Setting

The study was conducted in the lounge room of the Department of Community Emergency Health & Paramedic Practice simulation flat, a replica of a complete flat where various prehospital simulations are conducted.

### Participants

Students were eligible to participate in the study if they were enrolled in 2nd year of the Bachelor of Emergency Health (Paramedic) (BEH) or 3rd year of the Bachelor of Emergency Health (Paramedic)/Bachelor of Nursing (BEH/BN) course at Monash University, VIC, Australia. There were 119 students eligible to participate in the study.

All students had successfully completed resuscitation training, including automatic and manual defibrillation, in 1st and 2nd year for the BEH students and 2nd and 3rd year for the BEH/BN students. This training in in line with Ambulance Victoria requirements for paramedics when managing a cardiac arrest.

### Study size

A convenience sample of students was obtained through an announcement at the end of a lecture several days prior to the study. Participation was voluntary and no further announcements were made prior to the study.

### Equipment

#### Mannequin

The mannequin was a Laerdal VitalSim Resusci Anne^®^ which used a wireless control to manage the rhythms.

#### Monitor/defibrillator

The Phillips Heartstart MRx^®^ monitor/defibrillator was used for all simulations. The students had used this device during their training and were able to use it in automatic external defibrillator (AED) mode and full manual mode.

### Procedures

Students read a study explanatory statement and signed a consent form prior to participation in the study. Students were advised they could withdraw at any time from the study without penalty and that the data collected would in no way identify them.

Each student completed two 10-min cardiac arrest simulations with each cardiac arrest simulation requiring three defibrillation attempts. The student had a partner who for the simulation was only required to do as they were asked by the student being assessed.

In the first cardiac arrest simulation the patient remained in a shockable rhythm for the duration of the simulation and required a three defibrillation attempts separated by 2 min of cardio-pulmonary resuscitation (CPR).

In the second cardiac arrest simulation there were additional issues to deal with. There was loud music when the crew entered the room, there was an uncovered needle attached to a syringe on the edge of a coffee table next to the patient, and a disruptive bystander to manage. The bystander was constantly trying to touch the simulated patient which the student had to manage. In this simulation the patient underwent rhythm changes following the first defibrillation attempt, the second rhythm was a pulseless ventricular tachycardia (VT) which reverted back to ventricular fibrillation (VF) following the second defibrillation attempt and remained in that rhythm for the remainder of the simulation.

In line with their education, students commenced the resuscitation with the monitor/defibrillator in AED mode and after the first defibrillation switched to manual mode from which all further shocks were delivered. The Phillips Heartstart MRx^®^ uses pads in AED and manual mode.

### Instrumentation

Each student rated their safety aspects during the cardiac arrest simulation using a defibrillation safety self-assessment (DSSA) form immediately after completing both the cardiac arrest simulations.

The students had to answer yes or no to questions on the DSSA about their verbalising commands, making eye contact with their partner, and visually scanning the scene before analysing, charging the defibrillator and shocking the patient.

The student’s performance was rated using the DSSA by an independent Faculty staff member during each simulation.

### Outcomes

The first set of outcomes were whether the student verbalised “stand clear” prior to the defibrillator analysing the cardiac rhythm, prior to charging the defibrillator, and prior to “shocking” the patient. The second set of outcomes were whether the student made eye contact with their partner prior to the defibrillator analysing the cardiac rhythm, prior to charging the defibrillator, and prior to “shocking” the patient. The final set of outcomes were whether the student made a visual scan of the immediate area prior to the defibrillator analysing the cardiac rhythm, prior to charging the defibrillator, and prior to “shocking” the patient.

### Data Analysis

The SPSS program (Statistical Package for the Social Sciences Version 22, IBM Corporation, Armonk, NY, USA) was used to analyse the data. Descriptive statistics including means, standard deviation (SD) and medians were used to summarise the demographic data. Inferential statistics, Cohen’s Kappa test of agreement, was used to compare the level of agreement between student and assessor for each task required during the two simulations. All tests were two tailed with the results considered statistically significance if the *p* value is <0.05.

### Ethics

Ethics approval for the study was granted by the Monash University Human Research Ethics Committee.

## Results

### Participant demographics

There were 24 of 119 (20 %) eligible students who participated in the study. Of the 24, 14 (58 %) of the participants were female. Eleven students (46 %) were in 2nd year of the BEH, with the remainder in 3rd year of the BEH/BN. The average age of the students was 24.1 years with a SD of 5 years, median age was 21.5 years.

There were no inadvertent shocks received by the partner in either scenario or the bystander in scenario two.

#### Simulation 1

There was statistically significant agreement between the student and assessor for a visual scan of the scene during charging of the defibrillator for all three defibrillation attempts, with the agreement ranging from 29 % (p = 0.044) to 36 % (p = 0.021). There was also statistically significant agreement for verbalising “all clear” just prior to initiating a shock for defibrillation attempt one, 46 % (p = 0.007) and defibrillation attempt three where there was total agreement (p < 0.0001), see Table 1.

**Table 1:** Comparison of results for the student and assessor for simulation one

	Verbalise	Eye contact with partner	Visual scan of scene
Defibrillation 1			
“Stand clear” analysing	K = 0.229 p = 0.247	K = −0.083 p = 0.537	K = 0.0 p = 1.0
“Stand clear” charging	K = −0.212 p = 0.235	K = 0.93 p = 0.477	K = 0.364 p = 0.021
“All clear” shocking	K = 0.467 p = 0.007	K = 0.229 p = 0.085	K = 0.071 p = 0.572
Defibrillation 2			
“Stand clear” analysing	K = 0.083 p = 0.653	K = 0.185 p = 0.118	K = 0.0 p = N/A
“Stand clear” charging	K = 0.067 p = 0.772	K = 0.245 p = 0.132	K = 0.289 p = 0.044
“All clear” shocking	K = 0.0 p = 1.0	K = 0.169 p = 0.35	K = 0.077 p = 0.327
Defibrillation 3			
“Stand clear” analysing	K = 0.034 p = 0.851	K = −0.124 p = 0.295	K = −0.084 p = 0.455
“Stand clear” charging	K = −0.2 p = 0.285	K = 0.214 p = 0.09	K = 0.289 p = 0.044
“All clear” shocking	K = 1.0 p < 0.0001	K = 0.0 p = 1.0	K = 0.005 p = 0.959

#### Simulation 2

There was statistically significant agreement between the student and examiner for a visual scan of the scene during charging of the defibrillator for defibrillation attempt one, 29 % (p = 0.004) and defibrillation attempt two, 42 % (p = 0.025). There was also statistically significant agreement for having eye contact with their partner just prior to initiating a shock for all three defibrillation attempts, with the agreement ranging from 40 % (p = 0.043) to 53 % (p = 0.003). There was a statistically significant agreement for verbalising “all clear” just prior to initiating a shock for defibrillation attempt one, 65 % (p = 0.001), see Table 2.

**Table 2:** Comparison of results for the student and assessor for simulation two

	Verbalise	Eye contact with partner	Visual scan of scene
Defibrillation 1			
“Stand clear” analysing	K = −0.013 p = 0.939	K = −0.024 p = 0.803	K = 0.0 p = N/A
“Stand clear” charging	K = −0.135 p = 0.449	K = 0.196 p = 0.107	K = 0.289 p = 0.044
“All clear” shocking	K = 0.647 p = 0.001	K = 0.4 p = 0.043	K = 0.077 p = 0.327
Defibrillation 2			
“Stand clear” analysing	K = −0.083 p = 0.653	K = −0.024 p = 0.803	K = −0.084 p = 0.455
“Stand clear” charging	K = −0.149 p = 0.343	K = 0.529 p = 0.003	K = 0.417 p = 0.025
“All clear” shocking	K = −0.043 p = 0.831	K = 0.129 p = 0.525	K = 0.111 p = 0.439
Defibrillation 3			
“Stand clear” analysing	K = 0.034 p = 0.851	K = −0.032 p = 0.742	K = 0.0 p = N/A
“Stand clear” charging	K = 0.111 p = 0.569	K = 0.462 p = 0.007	K = 0.25 p = 0.132
“All clear” shocking	K = N/A p = N/A	K = 0.2 p = 0.317	K = −0.024 p = 0.703

## Discussion

This study showed that students perceived performance during the cardiac arrest simulation did not correlate with what the assessor observed during the simulation. There was at best some moderate agreement between the student and assessor, however, the students in most cases failed to follow taught processes for defibrillation safety during the simulations. This lack of defibrillation safety has implications for future education in cardiac arrest simulations, and potential clinical practice as future practicing paramedics.

The lack of defibrillation safety has been identified with fulltime paramedics in a low-volume workload area. In a study by Nielsen et al. paramedics on Bornholm Island, just off Denmark, successfully meet 30 % of the required defibrillation safety components (Nielsen et al. 2012). This was attributed to a low cardiac arrest workload, approximately 50 per year, and a possible lack of quality continuation training in managing cardiac arrests (Nielsen et al. 2012). These findings from the Nielsen et al. study may be generalisable to paramedic students in this study because they have completed the cardiac arrest management in two semesters of the course and then they tend to concentrate more on other conditions and treatment regimes and do not keep reviewing cardiac arrest management.

There is a paucity of literature describing the incidence and outcomes of paramedics who received a shock during the defibrillation process. The study by Gibbs et al. (Gibbs et al. 1990) identified paramedics who received a shock during defibrillation with only one person requiring hospitalisation for 3 days. Gibbs and colleagues (Gibbs et al. 1990) also reported on 3 years of USA Food and Drug Administration data about injuries associated with defibrillation and found three paramedics were admitted to hospital for observation.

It has been common defibrillation safety teaching for many years to state “clear” loudly and seek confirmation everyone is clear before shocking the patient (Kerber 2008; Cook 2003). With the advent of AED defibrillators in the late 1980s lay rescuers are prompted by the defibrillator to ensure they are clear of the patient prior to pressing the shock button. The students in this study were educated to verbalise “stand clear” and seek conformation of “all clear” prior to shocking the patient. Even though the students first semester of cardiac arrest management is undertaken with an AED and their second semester of cardiac arrest management is undertaken with a manual defibrillator there is no excuse for a lack of scene safety. What this study has shown is that the majority of students perceive they are performing these tasks, when in fact assessor evaluation confirms they are not.

The students were poor at scanning the scene during most components of the cardiac arrest and may lack the use of “peripheral vision” and constant visual scanning due to a lack of experience. Kirby et al. found in a study of military helicopter pilots undertaking a high speed low level flight task that the more experience pilots covered more in the scanning of the instruments in less time, maintained a level flight altitude and spent less time looking outside for a long time compare to the novice pilots (Kirby et al. 2014). The issue may be that student paramedics are not completely familiar with managing a cardiac arrest scene so spend more time focusing on the defibrillator and concentrating on the tasks they need to perform and do not think about a constant visual “sweep” of the scene to ascertain what is happening, especially from a safety perspective.

The results from this study show that less than 50 % of the time the students were scanning the incident prior to defibrillation, which poses a potential major safety threat to other healthcare providers and bystanders. The second simulation had a bystander who was constantly trying to touch the patient, therefore it was crucial for students to be scanning the scene thoroughly to ensure the bystander was not touching the patient before shocking the patient. During the “shocking” of the patient if a person has contact with the patient there is a potential for the electrical current to pass through them, this may lead to adverse effects such as skin burns and potentially lethal heart arrhythmias (Gibbs et al. 1990; Kerber 2008). Scene scanning appears to be a skill that requires further emphasis during cardiac arrest training.

Inadvertent shock from defibrillation appears to be diminishing as a result of healthcare providers wearing gloves, as has been described by Kämäräinen and Virkkunen (Kämäräinen and Virkkunen 2009). Likewise a study by Lloyd et al. (Lloyd et al. 2008) confirms the relative safety of defibrillation during chest compressions. However a recent study by Lemkin et al. (Lemkin et al. 2014) suggests that the action of hands on defibrillation still posses an unacceptable risk to healthcare providers so it should be discontinued until further research is conducted on appropriate gloves and/or supporting equipment.

The issue of a simulation versus the “real world” could be raised, however, if the students perform so poorly at defibrillation safety in a simulation what will they be like at a real cardiac arrest with multiple distractions and environmental factors? Henneman et al. state that errors are common in a simulation setting as the participant is being more heavily scrutinised compared to the “real world” and hence more errors are identified (Henneman et al. 2010). Conversely, Fisher et al. who examined hazard identification and anticipation of drivers in a vehicle simulator found that the driver’s performance in the simulator mirrored how they would behave in the “real world”.

Further studies using video recording glasses would be beneficial so that students can see and hear what they are not doing in respect to defibrillation safety during a simulated cardiac arrest and other clinical aspects of prehospital care simulation. The use of video glasses will aid educators in providing factual feedback and thereby demonstrating what the student is not doing during a cardiac arrest and other clinical simulations.

This study is potentially limited by the small sample size and hence the students that participated in the study may not have been a true representation of all undergraduate paramedic and paramedic/nursing students at Monash University. As the study was conducted at only one institution in Australia that educates paramedics the results may not be a true reflection of all paramedic students across Australia.

## Conclusion

The results of this study suggest student perception of their performance and what they actually do is vastly different. The results of this study have provided faculty with evidence of student underperformance in relation to safety during clinical simulations and also provides solid evidence for curriculum quality assurance. Further studies using video recording glasses are required so that students can gain an accurate and realistic sense of their defibrillation safety performance.
